# Compartment Syndrome following Below-Knee Amputation

**DOI:** 10.1155/2022/1256823

**Published:** 2022-02-21

**Authors:** Adam S. Gerry, Zachary K. Christopher, Karan Patel, Todd A. Kile, Joshua S. Bingham

**Affiliations:** ^1^Midwestern University-Arizona College of Medicine, Glendale, Arizona, USA; ^2^Department of Orthopedics, Mayo Clinic in Arizona, Phoenix, Arizona, USA

## Abstract

In the setting of below-knee amputation, compartment syndrome is a rare complication. Early clinical symptoms of an acute compartment syndrome following below-knee amputation can mimic or be masked by postoperative pain management. We present the case of a 38-year-old male with a significant past medical history of Proteus syndrome who underwent an elective transtibial below-knee amputation. Following surgery, the patient had extensive postoperative pain and high pain medication requirements and returned to the operating room for irrigation and debridement due to suspicion of an infection. Upon return to the operating room to manage the infection, the necrotic tissue was discovered and removed which had developed due to a suspected missed acute compartment syndrome. The necrotic tissue secondary to the compartment syndrome subsequently resulted in infection. Multiple irrigation and debridement procedures were performed to further manage the infection, and ultimately, the patient was deemed stable for discharge. Acute compartment syndrome (ACS) following below-knee amputation (BKA) is a rarely documented but critical complication. This case describes the unique setting in which a compartment syndrome can be masked due to postoperative pain management and infection. Orthopedic surgeons should be aware of the varying risk factors and presentations of an acute compartment syndrome (ACS) as it can occur and is a devastating complication.

## 1. Introduction

Below-knee amputation (BKA) is a treatment option which can be performed to remove ischemic, infected, necrotic, or otherwise nonfunctional tissue from the lower extremity. Amputation techniques for a BKA procedure vary in location from posterior flap transtibial amputation to sagittal, skew, medial, and fish-mouth flaps. A randomized trial showed that no significant differences were found in rates of primary stump healing, postoperative surgical site necrosis, or mortality across the different amputation techniques [1]. Still, complications following lower extremity amputation are common due to the nature of the procedure. Ciufo et al., when reviewing data from the National Surgical Quality Improvement Program (NSQIP) from a sample of 4,631 below-knee amputations between January 2012 and December 2014, showed an overall major complication rate of 12.8% (*n* = 593) within 30 days. These major complications most frequently included organ/deep space surgical site infections (infections involving the deep soft tissues, or any part of anatomy manipulated other than the incision area), wound dehiscence, pulmonary embolism, deep vein thrombosis, and other vascular issues. In this cohort, 9.63% (*n* = 446) of patients underwent unplanned reoperations.

The most common of those unplanned reoperations were above-knee or transfemoral thigh amputations (28.7% of reoperations), debridement/secondary closure (25.6%), and revision leg amputations (10.32%) [2]. Further studies show that postoperative wound infection following a major lower extremity amputation has been seen in anywhere from 13-40% of cases [3–5]. However, compartment syndrome is not a well-established complication of below-knee amputation.

Compartment syndrome is a limb- and occasionally life-threatening condition caused by an increase in intracompartmental pressure which can compromise vascular perfusion and cause hypoxemia of tissues, leading to necrosis [6]. Acute compartment syndrome (ACS) is most frequently seen following a traumatic event but can occur in up to 30% of cases without fracture. Additionally, studies suggest that muscle necrosis is more commonly seen in acute compartment syndrome without a fracture compared to ACS in the presence of a fracture [7, 8]. Ultimately, ACS can develop as a result of any pathologic condition (including swelling and/or bleeding) that causes an increase in compartment pressure exceeding perfusion pressure of tissue without increasing the volume of the myofascial compartment [9].

We present a case in which a patient with a significant medical history of Proteus syndrome and congenital deformity underwent an elective below-knee amputation secondary to pain and difficulty managing, cleaning, and using modified footwear for the affected lower extremity. Following surgery, the patient had extensive postoperative pain and high pain medication requirements and developed a stump infection. Upon return to the operating room to manage the infection, irrigation and debridement was performed, and the necrotic tissue was discovered and removed which we believe had developed due to a missed ACS. The necrotic tissue secondary to the compartment syndrome was likely what led to the infection which was treated with multiple irrigation and debridement and antibiotics. This case demonstrates the uniqueness of a possible postoperative complication of below-knee amputation.

## 2. Case

A 38-year-old male with a past medical history of Proteus syndrome presented to the clinic with initial complaints of pain due to a congenital deformity of his right foot. The patient stated that he was born with a deformity of his right foot and had 3-4 surgeries on the extremity as an infant for which he could not provide medical records prior to emigrating to the United States. The patient had not undergone any further surgical treatment for his lower extremity. The patient was required to wear one larger shoe and make significant modifications to his shoes, but that did not provide full relief of his pain. The patient also had a history of ingrowing nails on the lesser toes of the right foot, which he had been managing at home. In addition, the patient described difficulty cleaning the folds of skin on the plantar aspect of the right forefoot and plantar lateral heel secondary to pain.

**Figure 1:**
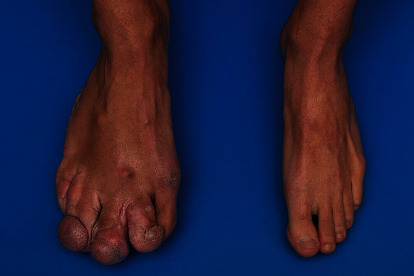
Photograph of the patient's bilateral dorsal feet, demonstrating the hypertrophy of digits 2, 3, and 4 and bony prominence over the right 3^rd^ metatarsal head.

**Figure 2:**
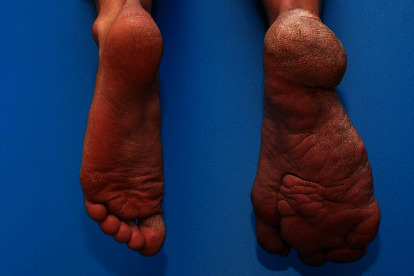
Photograph of the plantar surface of the patient's bilateral feet, demonstrating the multiple invaginations and skin folds on the plantar right foot.

**Figure 3:**
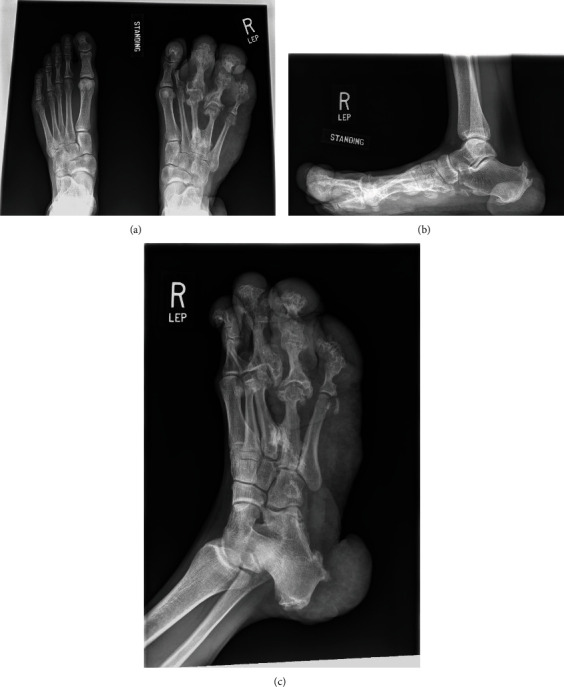
Anteroposterior, lateral, and oblique radiographs of the bilateral feet demonstrating macrodactyly of the right foot involving the second, third, fourth, and fifth toes with medial deviation of the second ray, pseudoarticulation of the third and fourth metatarsals, and advanced degenerative changes of the third and fourth metatarsophalangeal joints and interphalangeal joints of the second through fifth toes. Lateral view demonstrates soft tissue enlargement of the calcaneal soft tissues and large plantar and posterior calcaneal enthesophytes.

On initial examination and shown in Figures 1 and 2, the patient was shown to have significant hypertrophy of digits 2, 3, and 4 of the right foot with multiple invaginations and skin folds on the lateral border of foot and lateral soft tissue of the heel. While the great toe remained relatively well-preserved and normal, the patient displayed a bony prominence over the right 3^rd^ metatarsal head with impending skin breakdown. Additionally, there was hypertrophy of the plantar soft tissues beneath the lesser metatarsal heads 3-5 and hypertrophy of the affected calf when compared to the opposite. The remainder of the lower extremity exam was normal with intact symmetry of the legs and palpable pedal pulses bilaterally. Weightbearing radiographs of the right foot (Figures 3(a), 3(b), and 3(c)) confirmed hypertrophy of the 3^rd^, 4^th^, and 5^th^ rays including the phalanges with significant soft tissue hypertrophy at the lateral border of the foot and lateral heel with multiple exostoses/enthesophytes. As the patient had difficulty managing his right lower extremity for the majority of his life, treatment options were discussed with the patient to include amputation of the right lesser toes through the metatarsophalangeal joints vs. right lower extremity transtibial below-knee amputation and subsequent fit for prosthesis. The patient met with a prosthetist to undergo preamputation consultation and to attend an amputee support group meeting to provide additional insight and assistance prior to making his decision. The patient elected to proceed with transtibial BKA.

Roughly four weeks after initial consultation, the patient was admitted to the hospital and underwent successful right lower extremity transtibial BKA with a posterior flap under general anesthesia with a surgeon-placed trans biceps popliteal nerve block. This block consisted of 15 cc 1.5% mepivacaine and 15 cc of 0.5% ropivacaine. During the procedure, the anterior cut was marked approximately 10 cm below the tibial tuberosity with the posterior flap extending 150% the length of the anterior flap to this to provide adequate coverage for the below-knee amputation stump without significant tension. A rasp was used to smooth the edges of the cut bone, and bone debris was meticulously irrigated out of the wound. All major vessels were tied including the anterior and posterior tibial vessels as well as the peroneal vessels. The superficial peroneal, deep peroneal, and tibial nerves were pulled with tension and amputated proximally with cautery. A myodesis was performed by suturing the posterior compartment musculature overlying the distal stump of the tibia and fibula. No drain was utilized. Sterile dressings were applied followed by intraoperative fitting in creation of a “stump shrinker” and “stump protector” by a prosthetics service company. The patient was awakened and brought to the recovery area in stable condition and denied pain.

Roughly 9 hours following the patient being brought to the recovery area, the patient described severe pain, up to a 10/10 rating thought to be rebound pain from the block wearing off. This pain was uncontrolled with both PO and IV medications. Staff from the pain medicine team were consulted and saw the patient on postoperative day #1 (POD#1), where the patient described his pain as “nervy, crampy, and sharp,” and stated his pain was interfering with sleep. Pain was better controlled into the evening of POD#1 with PO and IV scheduled and as-needed analgesic medication. On POD#2, an orthopedic surgical resident evaluated the incision site to show a small area of necrosis anteriorly and a firm stump. The limb was moderately swollen, but the compartment remained compressible. The patient's vital signs were stable overnight with mild hypertension noted approximately 160 mmHg systolic and 70 mmHg diastolic. Labs were noted for an elevated creatine kinase (CK) of 10,013 (normal lab value 55 to 170 units/L [10]), and the patient had continued pain with subjective ratings from 8-9/10 throughout the evening. The patient continued to deal with poorly controlled pain for several days despite involvement of pain medicine until he was deemed stable for discharge on POD#4.

**Figure 4:**
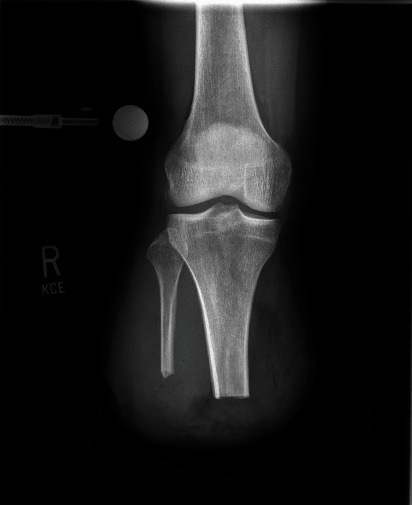
Anteroposterior radiograph of the right knee fourteen days postoperatively showing transtibial BKA.

At the patient's one-week follow-up status post transtibial BKA, he continued to have pain control issues and on exam showed a small area of bloody drainage from the central to lateral portion of the wound with resolving ecchymosis and moderate swelling throughout the stump. At a visit 11 days status post BKA, the patient was prescribed a course of Bactrim DS 1 po BID due to suspicion for infection after noting erythema just distal to the knee with warmth at the surgical site on exam. At another visit 14 days postoperatively, the patient reported that his pain was slowly improving but stated that he had self-recorded a low-grade fever of 100.3 degrees at home. On exam, there was a significant amount of active drainage on the dressing, continued swelling, mild erythema proximal to the incision, and warmth to the touch just distal to the knee. Deep cultures were taken and eventually showed growth of *Enterobacter cloacae* resistant to augmentin, ampicillin, and cephazolin. Laboratory bloodwork showed a sedimentation rate of 71 mm/hr, CRP of 142.3 mg/dL, and WBC of 18.3 cells/*μ*L (normal values are 0-15 mm/hr, ≤0.8 mg/dL, and 0-5 cells/*μ*L, respectively [10]). Radiographs were taken on postop day 14 and are shown in Figure 4. Patient was directly admitted to the hospital on this same day for a postoperative infection.

**Figure 5:**
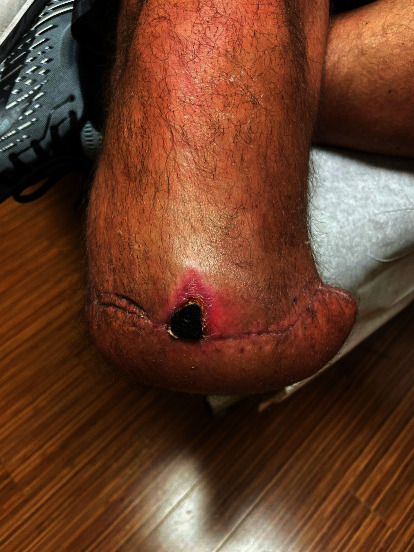
Photograph of the right lower extremity stump 77 days following the initial right transtibial BKA.

The patient returned to the operating room for irrigation and debridement of the surgical site 17 days following his BKA procedure. During surgery, the previous surgical incision was utilized removing all sutures in skin as well as deeper sutures from the previous surgery. Purulence was noted on entering previous surgical incisions, and cultures were taken from the superficial fluid. On exposing the deep compartments of the leg, gross purulent fluid and necrotic muscle were noted most prominently in the lateral compartment of the stump. This area extended anterior, lateral, and posterior to the fibula. Purulence and necrotic tissue were debrided to a bleeding border with curettes, rongeurs, and with sharp and blunt dissection. Following this surgery, the patient had a similar postoperative course to the first and had continued difficulty with pain management. The surgical wound had persistent drainage with concern for ongoing infection due to the volume and change in color of drainage from a bright red to a darker red with brown tinge. The patient underwent a repeat surgery three days following the first irrigation and debridement to manage the infection. The second irrigation and debridement procedure showed progression of the infection into the deep posterior and lateral compartments of the leg despite prior thorough debridement and IV antibiotic therapy. There was a significant amount of necrotic muscle and deep tissue from the posterior compartment of the leg which was debrided thoroughly. At this juncture, negative pressure therapy was employed to aid in wound management. Cultures taken during surgery confirmed growth of *E. cloacae*, and the patient continued to be followed closely by infectious diseases. After another four days, the patient was returned to the OR for a third irrigation and debridement of the wound which showed improved condition of muscle and other soft tissues. Much of the surgical site was shown to have healthy, bleeding tissue. There was a single remaining pocket with purulence in the anterolateral leg which extended proximally. The patient continued to have difficulty with pain management during this postoperative course. Finally, a fourth irrigation and debridement procedure was performed in the OR 27 days following the initial surgery. At this time, the subcutaneous and deep tissue had no sign of gross infection and with good granulation tissue. The site was irrigated as it had been previously with double antibiotic solution and sterile saline, and the patient was deemed stable and was discharged from the hospital the following day. The remainder of the postoperative course was uneventful, progress shown in Figure 5, and the patient has now been fit for a suitable prosthetic.

As evidenced by the presence of extreme pain immediately postoperatively, extensive pain medication requirements, elevated creatine kinase, and soft tissue necrosis noted in subsequent operations, this patient was diagnosed with a case of missed ACS and subsequent postoperative infection with *E. cloacae* following elective transtibial below-knee amputation. After multiple irrigation and debridement procedures to remove the necrotic tissue, the patient was stable for discharge home and to begin ambulating with a prosthesis.

## 3. Discussion

Compartment syndrome following below-knee amputation is a rare complication, and after extensive research, we believe that this is the first described case of this complication occurring. There are, however, notable reported cases of compartment syndrome following a free fibula osteocutaneous flap as a donor site in mandibular reconstruction [11–15]. While both procedures involve a significant bony lower leg amputation, the preoperative diagnoses and clinical reasoning for performing these surgeries are often very different. However, these cases highlight a critical possible complication of any procedure which involves amputation of any amount of bone.

While compartment syndrome most often appears following a traumatic event, it is important to note that ACS can occur following minor trauma or nontraumatic cases which cause an increase in intracompartmental pressure [7, 8]. ACS is seen more often in patients under 35 years old, and young men appear to have the highest incidence of cases [16], which may be due to an associated incidence of fracture injuries in this population. Moreover, the use of prophylactic anticoagulation following surgical procedures may potentially contribute to the development of ACS [17–19]. As well as compartment syndrome being seen in fibula flap harvests for oral maxillofacial grafts, cases have been reported following other lower extremity surgeries such as total knee arthroplasty and vein harvest for coronary artery bypass surgery [20–22]. Additionally, most symptoms of ACS can very closely mimic a normal postoperative course of pain, potentially delaying the diagnosis of compartment syndrome. Unfortunately, the typical moderate-to-severe pain often seen as a hallmark of early compartment syndrome can be masked by regional nerve blocks or perioperative anesthesia and postoperative analgesics. There are numerous reports of missed or delayed compartment syndrome associated with various forms of postoperative analgesia in literature [23–28]. In this patient's case, attempted management of pain with the involvement of the pain medicine team proved to be a significant challenge but was eventually ameliorated with a combination of oral and IV pain medications. While it is a rare complication, it is crucial that medical professionals are aware of this potentially catastrophic outcome.

Diagnosis of compartment syndrome requires a crucial suspicion of the disease when several factors are noted—severe pain, paresthesias, paralysis, and visible swelling of the compartment with a tense, “woody” feeling on palpation [29]. In addition, a creatine kinase level greater than 4,000 U/L has been shown to be associated with compartment syndrome [30], and our patient displayed a CK of 10,013 U/L on postoperative day two. However, it should be noted that elevations in creatine phosphokinase have been demonstrated in a dose-dependent relationship with the extent of surgical invasiveness [31], so it is possible that this increase was a consequence of the major surgery performed. Diagnosis of compartment syndrome when suspected can be confirmed by direct measurements of compartment pressures [32], but this was not performed in this case. It is possible that our patient developed a compartment syndrome postoperatively due to bleeding, as hemorrhage or hematoma can cause compartment syndrome in the absence of acute trauma [33, 34].

Our patient's medical history may have also played a role in the progression of his case. In a study reviewing 22 current Proteus syndrome patients and 100 previously reported on Proteus syndrome patients, Hoeger et al. found vascular anomalies in 22/22 of their current patients and reported anomalies in 70/100 of the previous patients [35]. It is plausible that the prevalence of vascular anomalies in this patient population could have played a role in the rise in intracompartmental pressure seen in this case.

Medical and nursing staff should be aware of the potential varying presentation of compartment syndromes, especially postoperatively where use of analgesics could mask certain symptoms. Additionally, this case describes an important caveat in describing compartment syndrome developing even after most of the lower leg was removed. These remaining tissues and compartments are still susceptible to bleeding, increased pressures, and potentially an acute compartment syndrome.
